# Real-time vehicle target detection in inclement weather conditions based on YOLOv4

**DOI:** 10.3389/fnbot.2023.1058723

**Published:** 2023-03-09

**Authors:** Rui Wang, He Zhao, Zhengwei Xu, Yaming Ding, Guowei Li, Yuxin Zhang, Hua Li

**Affiliations:** ^1^School of Computer Science and Technology, Changchun University of Science and Technology, Changchun, Jilin, China; ^2^Department of Geophysics, Chengdu University of Technology, Chengdu, Sichuan, China

**Keywords:** target detection, YOLOv4, inclement weather conditions, Anchor-free, Decoupled head

## Abstract

As a crucial component of the autonomous driving task, the vehicle target detection algorithm directly impacts driving safety, particularly in inclement weather situations, where the detection precision and speed are significantly decreased. This paper investigated the You Only Look Once (YOLO) algorithm and proposed an enhanced YOLOv4 for real-time target detection in inclement weather conditions. The algorithm uses the Anchor-free approach to tackle the problem of YOLO preset anchor frame and poor fit. It better adapts to the detected target size, making it suitable for multi-scale target identification. The improved FPN network transmits feature maps to unanchored frames to expand the model's sensory field and maximize the utilization of model feature data. Decoupled head detecting head to increase the precision of target category and location prediction. The experimental dataset BDD-IW was created by extracting specific labeled photos from the BDD100K dataset and fogging some of them to test the proposed method's practical implications in terms of detection precision and speed in Inclement weather conditions. The proposed method is compared to advanced target detection algorithms in this dataset. Experimental results indicated that the proposed method achieved a mean average precision of 60.3%, which is 5.8 percentage points higher than the original YOLOv4; the inference speed of the algorithm is enhanced by 4.5 fps compared to the original, reaching a real-time detection speed of 69.44 fps. The robustness test results indicated that the proposed model has considerably improved the capacity to recognize targets in inclement weather conditions and has achieved high precision in real-time detection.

## Highlights

- An enhanced YOLOv4 with Anchor-free and Decoupled head is proposed.
- Improved FPN enhances multi-scale feature fusion capabilities and thus detection performance.
- The proposed method is used to solve vehicle target detection in inclement weather conditions.
- Average accuracy has been improved by 5.8% and detection speed by 4.5 fps.

## 1. Introduction

With the rapid changes in market demand and the growth of the automobile industry, autonomous driving has become one of the most researched topics in the automotive industry. In the case of autonomous driving, road safety depends significantly on identifying impediments ahead. Target detection, the most fundamental part of autonomous driving, collects real-time environmental data for the vehicle to assure safety and make sound planning judgments. Especially in inclement weather circumstances such as rain, snow, and fog, the detection precision and speed are significantly impacted by other elements such as fog, increasing the likelihood of automobile accidents. Consequently, more precise and quicker target detection technologies are required to lower the danger of pedestrian and vehicle collisions under inclement weather conditions. In target detection tasks, inclement weather conditions can significantly impact the performance of image and video-based traffic analysis systems (Hamzeh and Rawashdeh, 2021). Histograms of Oriented Gradients (Arróspide et al., 2013), the Deformable Part Model (Cai et al., 2017), the Viola-Jones (Xu et al., 2016), and so forth. Traditional target detection methods cannot match the requirements for rapid and precise recognition of low-quality pictures.

In addition, most previous methods for detecting targets emphasize specific resource needs. Despite this, many real-world applications (from mobile devices to data centers) frequently have diverse resource limits. Computation platforms for automated driving have constrained memory and computing resources. Consequently, the most recent trend in network model design should investigate portable and efficient network designs. Detection methods applied to in-vehicle platforms must have relatively minimal memory and computing resource footprints. Girshick et al. (2015) implemented Region-Convolutional Neural Networks (R-CNN) for target detection in 2014. Traditional target detection approaches are progressively losing ground to deep learning.

Target detection based on deep learning has demonstrated unique advantages in autonomous driving. This approach is crucial for autonomous driving systems because it can achieve high detection precision with fewer computational resources (He and Liu, 2021). The most frequent frameworks for target identification fall into two categories: R-CNN (van de Sande et al., 2011), Fast R-CNN (Girshick, 2015), and Faster R-CNN (Ren et al., 2017) and two-stage target detection algorithms. The first stage of the two-stage procedure is the generation of candidate boxes that contain both true and false target items for detection. The second stage involves analyzing the boxes and identifying the target within each box. The other single-stage target identification approach is You Only Look Once (YOLO) (Redmon et al., 2016), Single Shot MultiBox Detector (SSD) (Liu et al., 2016), etc. Even though single-stage target detection algorithms are somewhat less accurate than two-stage target detection algorithms in terms of detection accuracy, single-stage target detection methods are frequently employed in moving vehicle target detection (Chen et al., 2020; Zhou et al., 2020; Wu et al., 2021). Obviously, for detection precision concerns, increasingly efficient single-stage algorithms such as YOLOv3 (Redmon and Farhadi, 2018), YOLOv4 (Bochkovskiy et al., 2020), CenterNet (Zhou et al., 2019a), RetinaNet (Lin et al., 2020), etc. are being applied to driving scenarios. Researchers have attempted to use YOLOv4 and its enhancements in different fields since its introduction. Yu et al. (2021) introduced a deep learning model named YOLOv4-FPM based on the YOLOv4 model and used it for the real-time monitoring of bridge cracks, which improved the mAP by 0.064 compared to the classic YOLOv4 technique. Han et al. (2021) suggested a Tiny-YOLOv4 technique that combines the Self-Attention mechanism with ECA-Net (Effective Channel Attention Neural Networks) for insulator detection. The detection method's speed, precision, and complexity are greatly enhanced. YOLOv4 is also utilized in the inspection of vehicles (Yang et al., 2021; Mu et al., 2022).

Among these target detection approaches, YOLOv4 has been utilized in various fields due to its balance of speed and precision. However, the No Free Lunch Theorem (NFL) (Wolpert and Macready, 1997) demonstrates that no single method can effectively tackle all problems. Due to degraded image quality, YOLOv4's performance in predicting inclement weather conditions could be better. Moreover, although the little study has been conducted on vehicle target detection in inclement weather, vehicle detection in inclement weather cannot be overlooked in autonomous driving and autos. This paper provides a YOLOv4 enhancement approach to address the concerns mentioned above. The experimental results demonstrate that the detection model provided in this study can detect vehicle targets under inclement weather conditions.

Following are the paper's primary contributions: (1) An enhanced approach based on YOLOv4 is proposed. The approach introduces Anchor-free and Decoupled head (2) Enhancements to the FPN to improve the multi-scale feature fusion capabilities for improved detection performance and increased target detection accuracy. (3) The BDD100k dataset is extracted and processed to create the inclement weather vehicle detection dataset BDD-IW for the experiments presented in this research. (4) The proposed technology is applied to vehicle detection in inclement weather conditions. Compared to YOLOv4, the mean average precision is 5.8% higher, and the detection speed is 4.5 frames per s faster.

The following are the remaining sections of this work. A literature review is presented in Section 2. Section 3 provides a comprehensive explanation of the method proposed in this study. The findings of the tests done to validate the performance of the proposed model are discussed in Section 4. Section 5 includes a summary of the current work and an analysis of future work.

## 2. Related works

### 2.1. Target detection in inclement weather conditions

Inclement weather can reduce the camera imaging quality, affecting the accuracy and speed of target detection, which can cause serious adverse results on road safety.

Xu et al. (2016) based on Viola Jones algorithm for UAV image vehicle detection method to detect the road direction first for detecting the object direction sensitive problem, and then correct the detection direction according to the road direction to achieve higher detection efficiency and accuracy. Kuang et al. (2018) developed a multi-class vehicle detection system based on tensor decomposition for night vehicle detection. This method can successfully detect the categories of cars, taxis, buses and minibuses in night traffic images, and also realize effective detection for vehicles that are obscured and vehicles in rainy days. Zheng et al. (2022) proposed an improved Fast R-CNN convolutional neural network for dim target detection in complex traffic environments, replaced VGG16 in Fast R-CNN with ResNet, adopted the downsampling method and introduced feature pyramid network to generate target candidate boxes to optimize the structure of the convolutional neural network. Humayun et al. (2022) used CSPDarknet53 as the baseline framework, and achieved reliable performance for vehicle detection under scenarios such as rain and snow through space pyramid pool layer and batch normalization layer. Guo et al. (2022) first proposed a data set for vehicle detection on foggy highway, and then proposed a foggy vehicle detection model based on improved generative adversarial network and YOLOv4, which effectively improves vehicle detection performance and has strong universality for low-visibility applications based on computer vision. Samir et al. (2018) proposed a methodology for target detection during foggy days. The model employed convolutional neural networks for image removal and Fast R-CNN for target detection. Hassaballah et al. (2020) presented a robust vehicle detection method with a multi-scale deep convolution neural network and introduced a benchmark dataset for vehicle detection under adverse weather conditions, which improved the detection efficiency compared to some current advanced vehicle detection methods. You et al. (2020) proposed a lightweight SSD network algorithm and detected vehicle targets in complex weather environments with 3% improvement in detection accuracy and 20% improvement in detection speed. Ghosh (2021) proposed a multiple region suggestion network using Faster R-CNN for detection under different weather conditions and achieved excellent detection performance with an average accuracy of 89.48, 91.20, and 95.16% for DAWN, CDNet 2014, and LISA datasets, respectively. Wang K. et al. (2021) developed a denoising network based on rainfall characteristics and input the resultant denoised images into the YOLOv3 recognition model for synthetic and natural rainfall datasets. The modified photos strengthened the process of target detection. Khan and Ahmed (2021) developed a novel convolutional neural network structure for detecting vehicle road images limited by weather factors, and the detection speed was significantly improved. Ogunrinde and Bernadin (2021) used CycleGAN combined with YOLOv3 for the KITTI dataset to improve the detection efficiency of moderate haze images. Wang et al. (2022) proposed a vehicle detection method based on pseudo-visual search and the histogram of oriented gradients (HOG)-local binary pattern feature fusion, which achieved an accuracy of 92.7% and a detection speed of 31 fps. Guo et al. (2022) proposed a domain-adaptive road vehicle target detection method based on an improved CycleGAN network and YOLOv4 to improve the vehicle detection performance and the generalization ability of the model under low-visibility weather conditions. Tao et al. (2022) improved YOLOv3 based on ResNet, and the improved network reduced the difficulty of vehicle detection in hazy weather and improved the detection accuracy. Humayun et al. (2022) proposed an improved CSPDarknet53 network to enhance the detection precision of targets in the haze, dust storms, snow, and rain weather conditions during day and night.

Analyzing prior work on target detection in inclement weather allows us to draw the following conclusions: Among the techniques mentioned earlier are noise reduction of the dataset, the usage of datasets that aid increase prediction accuracy, and improved target identification techniques, among others. (1) Using the picture denoising method. This strategy generated higher-quality training data for target detection and enhanced prediction accuracy. However, the presence of halo distortions, color distortion, low contrast, and a lack of clarity may decrease the effectiveness of the target detection algorithm when utilizing data of inferior quality-synthesizing the dataset to mimic inclement weather input to the target detector for training purposes. This strategy effectively addressed the limited quantity and poor diversity of training data. Nevertheless, the procedure cannot ensure the authenticity of the resulting dataset and still requires many reviews. (2) Target detector improvement. Detection is intended to increase the precision of the weather forecast model under challenging settings. The generalizability of the model must also be considered in the study.

### 2.2. YOLO methods

Since its inception, the YOLO series of methods has been among the most sophisticated target detection techniques. In 2016, Redmon introduced YOLOv1 (Redmon et al., 2016), which has one feature in common with other target identification algorithms, such as R-CNN, Fast R-CNN, and Faster R-CNN. They constructed many candidate boxes containing genuine and fake target objects for detection. In a two-step procedure, the first stage entails developing the boxes, while the second stage entails processing the boxes and identifying their precise targets. In contrast, Joseph Redmon proposed the YOLO algorithm in 2015, which uses a single-stage target detection algorithm that combines two steps into one, directly transforming the problem of target border localization into a one-step regression problem processing and making significant advancements in algorithm detection speed. Redmon and Farhadi (2017) introduced YOLOv2 in 2017 on top of YOLOv1. YOLOv2 rebuilt the network's backbone with DarkNet19 and introduced enhancements such as Conv+BatchNorm.

**Figure 1:**
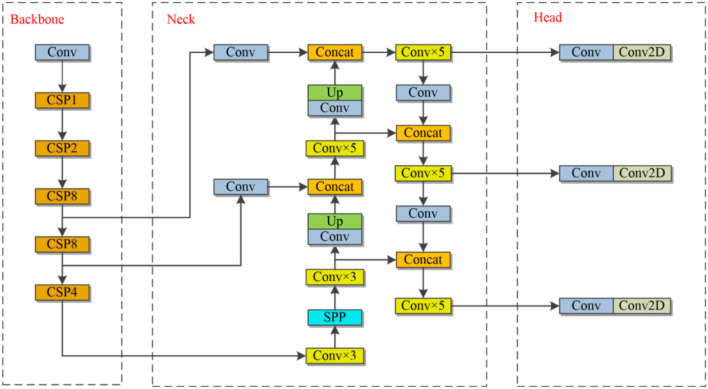
The structure of YOLOv4.

YOLOv3 (Redmon and Farhadi, 2018) also rebuilt the backbone network with DarkNet53 and added more improvements. Bochkovskiy et al. (2020) developed the Darknet framework and YOLOv4 based on the original YOLO hypothesis. With better processing, YOLOv4 was derived from YOLOv3. Extending the dataset while preprocessing the data delivers a performance boost to the network without the need for additional memory and model space, as well as a significant improvement in network detection at the expense of a slight decrease in speed. Ge et al. (2021b) suggested the YOLOX algorithm in 2021, based on the YOLO family, to obtain better and more meaningful outputs. The enhancement of this study is based on YOLOv4. The structure of the YOLOv4 network model can be broken down into three major components: the backbone, the neck, and the prediction head. Figure 1 depicts the network architecture diagram. The backbone network for extracting features, is the essential stage in acquiring the feature map. Visual Geometry Group (VGG) (Simonyan and Zisserman, 2014), Residual Network (ResNet) (He et al., 2016), ResNeXt (Xie et al., 2017), and Darknet53 (Redmon and Farhadi, 2018) are all conventional networks for feature extraction. Recent years have seen the emergence of a more deliberate Backbone. MobileNet (Howard et al., 2017) was an effective mobile and embedded applications architecture. EfficientNet (Tan and Le, 2019) enabled the scalability of models while preserving their precision and performance. HRNet maintained a high-resolution representation by simultaneously concatenating low- and high-resolution convolutions (Sun et al., 2019). The neck can achieve the merging of shallow and deep feature maps to make full use of the retrieved features by Backbone, from FPN to PANet (Liu et al., 2018), NAS-FPN (Ghiasi et al., 2019), BiFPN (Tan and Le, 2019), and so forth. Their relationships are growing progressively intricate. HRFPN (Sun et al., 2019) is a method of feature fusion proposed by HRNet to retain high resolution. Balanced Feature Pyramid takes advantage of balanced semantic features integrated at the same depth to improve multilevel features (You et al., 2020). It has been confirmed that the feature fusion structure provides a more significant boost to mAP.

The Head is primarily responsible for estimating the target's category and position. In order to locate the target's position, one uses the regression branch to localize the target's position and the classification branch to determine the target's class. However, the features emphasized during feature learning are distinct, causing the two branches to share most parameters and restricting the target identification effectiveness to some extent. The problem was initially discussed and investigated in 2018. Jiang et al. (2018) proposed an intersection over the union net (IoU-Net). IoU-Net implemented a new branch to anticipate IoU values as the confidence level for localization, and IoU-guided NMS was used to remove superfluous boxes. Wu et al. (2020) proposed Double-Head RCNN in 2020 and constructed it to implement classification and regression decoupling. Fully connected was deemed more appropriate for classification tasks, and convolution was deemed more suitable for regression tasks. Hence fully connected was used for classification branches, while convolution was utilized for regression branches. The dispute between classification and regression persists even though both branches received identical proposal characteristics after ROI pooling. In the same calendar year, Song et al. In the same year, Song et al. (2020) separated the classification and regression problems in target detection in terms of spatial dimensions. They noticed a misalignment between the classification and regression spatial dimensions. According to the article, TSD decouples classification and regression problems from the spatial dimension. Ge et al. (2021b) proposed YOLOX in 2021 to further upgrade to YOLO. One of the changes between YOLOX and earlier YOLO series algorithms was the decoupled head, which replaced the coupled detection head of YOLOv3 in the text.

The prediction of bounding boxes is also separated into two ways, one of which is anchor-based and the other of which is anchor-free. In recent years, anchor-free frame detection has become an alternative method for bounding-frame prediction, as the usage of anchor frames generates a significant imbalance in the number of positive and negative anchor frames, increases hyperparameters, and slows training. Anchor Free is now classified into the keypoint-based and center-based categories for describing detection frames. CornerNet (Law and Deng, 2018) and ExtremeNet (Zhou et al., 2019b) use the keypoint-based detection technique, which identifies the target's upper-left and lower-right corner points and then combines the corner points to produce a detection frame. FSAF (Zhu et al., 2019), FCOS (Tian et al., 2022), FoveaBox (Kong et al., 2020), and CenterNet (Zhou et al., 2019a) utilize center-based detection algorithms that directly detect the core region and border information of the object and decouple classification, action, and regression into two subgrids.

## 3. Materials and procedures

**Figure 2:**
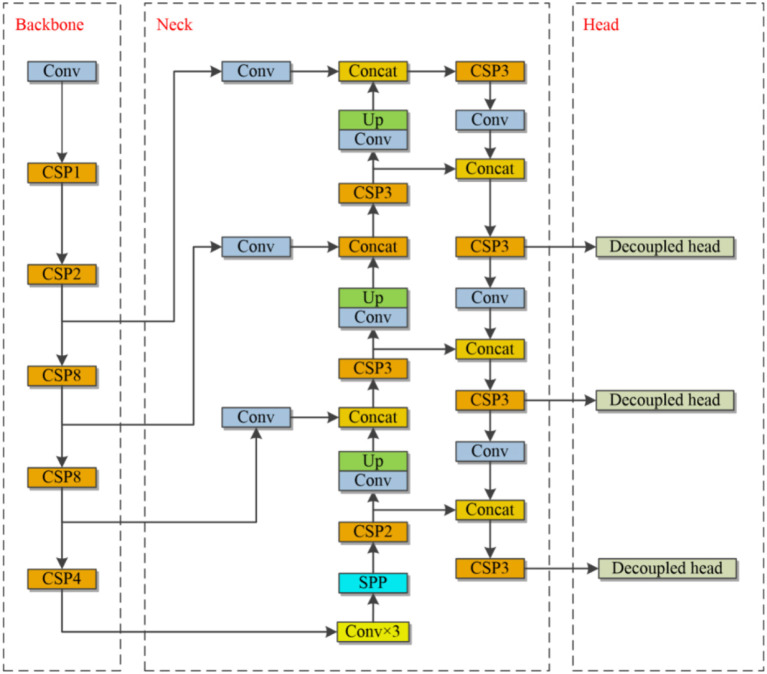
The structure of the proposed method.

This section explains the backbone network, the neck, the prediction head, and data enhancement in the YOLOv4-based enhancement model. Moreover, the BDD-IW dataset is described in depth. The pseudo-code of the proposed target detection algorithm is shown in Algorithm 1. Figure 2 depicts the network architecture diagram for the proposed method.

**Algorithm 1:** Pseudo-code of the proposed method.

1. Initialize network parameters such as learning rate, batch size, number of epochs
2. Load training data such as images, labels
3. Define loss function such as mean squared error, cross-entropy
4. Iteratively train network using backpropagation and gradient descent
5. Test network using validation data
6. Evaluate network performance using metrics such as precision, recall, mean average precision

### 3.1. Backbone

**Figure 3:**
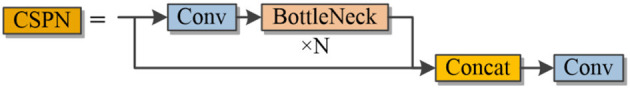
CSP in proposed work.

CSPDarkNet53 provides a good foundation for solving the feature extraction problem in most detection scenarios through the Cross Stage Partial (CSP) network. In order to simplify the network structure, this work uses a more straightforward and lighter CSP structure to replace the original method backbone network. Its structural function is exactly the same as the original CSP structure, but the correction unit consists of three standard convolutional layers and multiple bottleneck modules. In contrast, the Conv module is deleted after observing the residual output, and the activation function in the regular convolution module after concat is modified from LeakyRelu to SiLU. This module is the primary module for learning residual features and is divided into two branches, one of which uses the multiple Bottleneck stacks and three standard convolutional layers described above. The other undergo merely a single convolutional module. Lastly, the two branches are concat-operated. Figure 3 illustrates the CSP module's structure.

### 3.2. Neck

#### 3.2.1. Spatial pyramid pooling

**Figure 4:**
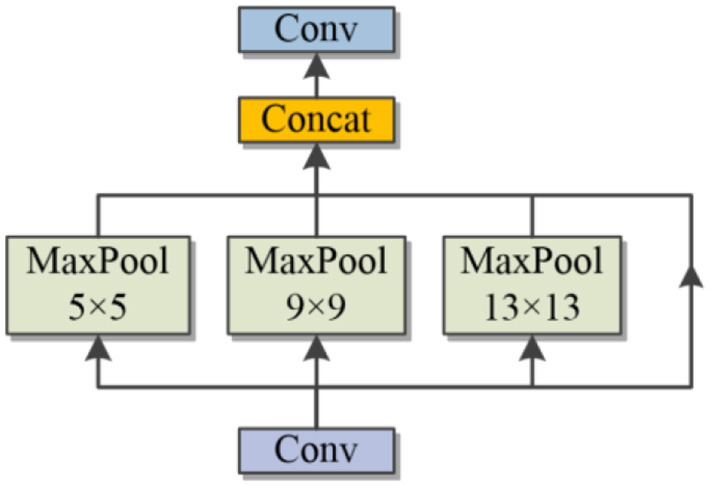
SPP layer.

In order to extract spatial feature information of different sizes, the robustness of the model for spatial layout and object denaturation is improved. In this study, the original method of spatial pyramid structure (SPP) was used, and the structure is shown in Figure 4.

#### 3.2.2. Improved feature pyramid network

**Figure 5:**
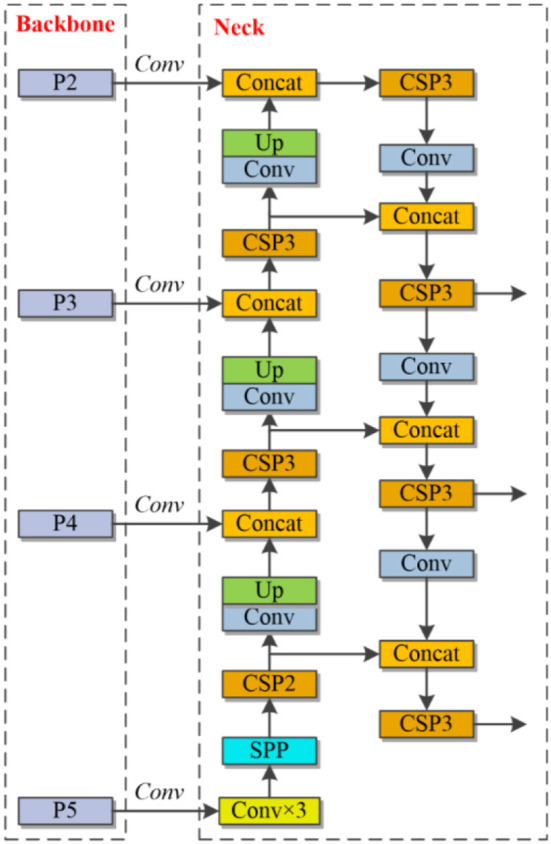
FPN in the proposed work.

FPN transmits semantic features from top to bottom and merges the information using upsampling procedures to predict results. In the original method, the prediction ability of the network is poor when the target in the image is too small, and the ability of the network to detect the target is greatly reduced by the lack of feature information after the convolution and downsampling process. To solve the above problems, this study obtains a feature layer with a narrower perceptual field containing more image-specific information based on the output of the second residual block feature layer of the backbone network. The lack of information in the low-level features of the original network for small-scale targets and the lack of information in the high-level feature layer are also compensated. At this stage, an enhanced FPN module is generated by extracting the output of the feature map of the second remaining module and fusing the other three feature layers together from shallow to deep. Figure 5 demonstrates the structure.

### 3.3. Head

By switching from the YOLO head to Decoupled head and utilizing the Anchor-free method for prediction, the prediction has been enhanced compared to the classic YOLO series.

#### 3.3.1. Decoupled head

**Figure 6:**
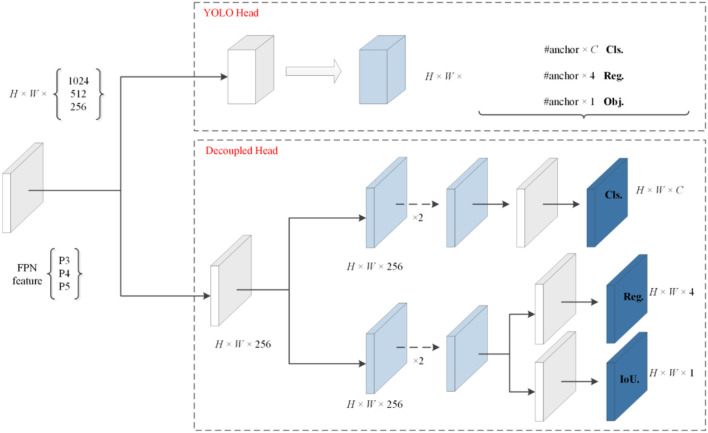
Decoupled head layer.

Their detection heads are coupled despite improving the YOLO series' backbone and feature pyramids. By replacing the YOLO head with a Decoupled head comprising two parallel branches, the heads of the classification and regression tasks no longer share the same parameters, and the convergence speed is significantly increased. There is a total of three concat branches in the output of the Head. The branch begins with a 1 x 1 convolution to accomplish channel reduction, followed by two more branches. The classification branch focuses mainly on the category prediction of the target box, while the other is the regression branch. The regression branch is subdivided into Reg and IoU to determine the target box location and IoU prediction. Then, the reshape operation is performed for the three pieces of information, followed by a global concat to obtain the complete prediction information. Figure 6 depicts the decoupled head construction.

#### 3.3.2. Anchor-free

To improve speed while maintaining precision. This approach shifts from anchor-based to anchor-free depending on the real-time identification of vehicle targets. YOLOv3 through YOLOv5 rely on anchors. However, anchor-based has issues with designing anchor points in advance, actively sampling images, and too much negative sample data. In this study, the anchor-free model reduces the number of predictions required per location from three to one. Allow them to forecast both the upper left corner offsets and the height and breadth of the prediction box. In addition, the 33 regions of the grid where each object's center is located are designated as a positive sample. A standard point range is specified to determine each object's FPN level. The method improves the speed and performance of detector delivery.

#### 3.3.3. Label assignment

During the training phase, label assignment is primarily the method by which the detector distinguishes between positive and negative samples and assigns a suitable learning target to each place on the feature map. Each feature vector contains the expected frame information when the number of anchor frames is collected. Primarily, Labeling the boxes within the positive sample anchors mainly filters them.

The initial screening is based on the center point and the target box. According to the target box method, the center of the anchor box falls within the rectangle of artificially labeled boxes (ground truth boxes) for all anchors that may be used to predict positive samples. According to the center point method, Using the center of the ground truth boxes as the base, expand the stride 2.5 times outward to form a square 5 times the length of the stride. All anchor boxes whose center points fall within the square may be used as predictions for positive samples.

This study introduces the SimOTA (Ding et al., 2021) algorithm for dynamically assigning positive samples to increase detection precision to obtain more high-quality positive samples. The approach for label assignment in YOLOv5 is based on shape matching. Cross-grid matching increases the number of positive samples, allowing the network to converge quickly. Nonetheless, this static allocation does not adapt as the network is taught. Recently, dynamic tag assignment-based techniques have also evolved. These methods award positive samples depending on the network's output during training, providing more high-quality examples and promoting the network's positive optimization. By modeling sample matching as an optimal transmission problem, OTA (Ge et al., 2021a) discovered the optimal sample matching approach using global information to increase precision. However, OTA took longer to train due to using the Sinkhorn-Knopp algorithm. The SimOTA algorithm uses the Top-K approximation strategy to get the best match for the samples, greatly speeding up the training. SimOTA first calculates the pairwise match, expressed as the cost or the quality of each predicted ground truth pair. For example, the cost between ground truth *g*_*i*_ and prediction *p*_*j*_ in SimOTA is shown in Equations (1)-(3).


(1) $$\begin{array}{l} c_{ij}=L_{ij}^{cls}+\text{λ}L_{ij}^{reg} \end{array}$$

(2) $$\begin{array}{l} L_{reg}=-log\left(\text{IoU}\left(B_{gt},B_{pred\;}\right)\right) \end{array}$$

(3) $$\begin{array}{l} L_{cls}=-\overset{n}{\underset{i=1}{\sum }}\left(t_{i}\log\left(p_{i}\right)+\left(1-t_{i}\right)\log\left(1-p_{i}\right)\right) \end{array}$$

Where λ is the equilibrium coefficient, and $L_{ij}^{cls}$ and $L_{ij}^{reg}$ are the classification loss and regression loss between *g*_*i*_ and *p*_*j*_. *L*_*reg*_ is determined using binary cross entbox (BCE). Bounding box regression loss *L*_*reg*_ using efficient intersection over union loss. *B*_*gt*_ and *B*_*pred*_ are the true frame and the predicted frame, respectively. *p*_*i*_ is the predicted value, and *t*_*i*_ denotes the true value of the predicted bounding box.

### 3.4. Data augmentation

An efficient data augmentation approach is employed to increase the training data to compensate for the training set's lack of images. The strategy uses the Mosaic and Mixup data augmentation methods and does not perform the method in the last 15 epochs. The Mosaic and Mixup data augmentation methods are described in detail in this subsection.

#### 3.4.1. Mosaic

In inclement weather conditions, vehicle detection is quickly impacted by fog, rain, and snow, which diminishes the precision of target recognition. The benefits of Mosaic data improvement are twofold. First, random cropping is used to enrich the local target features in the dataset, which is advantageous for model learning; Second, random stitching is used to retain all of the target features of the image without discarding the cropped features, and all of the image's features are utilized by the stitching method. This study incorporates Mosaic data augmentation during model training to increase the model's capacity to recognize veiled targets. Before each iteration begins, the proposed model reads images from the training set and generates new images through Mosaic data augmentation. The freshly generated images are mixed with the read images to create training samples, which are then fed into the model for training. The Mosaic data augmentation chooses four photos at random from the training set. Four pictures are randomly cropped and then stitched together to create a new image. The enhanced Mosaic data produces images with the same resolution as the four photos in the training set. Random cropping may remove a section of the target frame of the training set, replicating the effect of a vehicle target subjected to fog, rain, snow, or weak light.

#### 3.4.2. Mixup

Mixup is an image mixed class augmentation method that can expand the training data set by mixing images between different classes. Assuming that *x*_1_ and *x*_2_ are two batch samples, *y*_1_ and *y*_2_ are the labels corresponding to *x*_1_ and *x*_2_ samples, respectively, and λ is the mixing coefficient calculated from the beta distribution with parameters α, β, the mathematical model of Mixup is shown in Equations (4)–(6).


(4) $$\begin{array}{l} \text{λ}=Beta\left(\alpha ,\beta \right) \end{array}$$

(5) $$\begin{array}{l} x=\text{λ}x_{1}+\left(1-\text{λ}\right)x_{2} \end{array}$$

(6) $$\begin{array}{l} y=\text{λ}y_{1}+\left(1-\text{λ}\right)y_{2} \end{array}$$

where, *Beta* denotes a beta distribution, *x* is the blended batch sample, and *y* is the label corresponding to the blended batch sample.

### 3.5. Dataset

**Figure 7:**
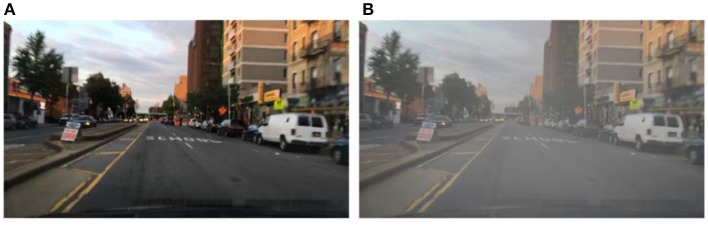
**(A)** Original case. **(B)** Fogging case.

**Figure 8:**
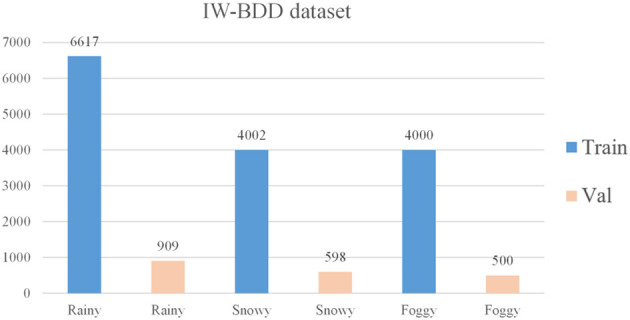
The distribution of weather labels.

There are 70,000 training sets, 10,000 validation sets, and 20,000 test sets in the BDD100k dataset (Yu et al., 2020). It covers various weather conditions, such as sunny, overcast, and rainy days, as well as various times of day and driving scenarios during the day and night. There are 10 ground truth box labels: person, rider, car, bus, truck, bike, motor, traffic light, traffic sign, and train. 7:1 is the ratio between the training set and the validation set. There are around 1.46 million object instances in the training and validation sets, of which ~800,000 are car instances, and just 151 are train instances. This disparity in category distribution reduces the network's ability to extract features. Hence train, rider, and motor are disregarded in the final evaluation. The completed BDD100k dataset includes seven categories: person, car, bus, truck, bike, traffic signal, and traffic sign. In the BDD100k dataset, each image has a different weather label. Since this research solely examines the differences between models under inclement weather conditions, the images with weather labels of rain and snow are extracted from the training and validation sets. And some images with weather labels as sunny are extracted, and fog processing is applied to these images to form a hazy sky scene, as shown in Figure 7. The RGB channel of the image is processed, setting the brightness to 0.6 and the fog concentration to 0.03. Finally, the images with the three weather labels of sunny, rainy, and snowy days processed by adding fog are counted to form the dataset BDD-IW for this research training, the distribution of weather labels in the dataset is shown in Figure 8. The format of the BDD-IW dataset is divided into two folders: training and validation sets. Each folder contains two more folders that store the images in JPG format and the corresponding labels in TXT format for each image. The final dataset consists of 14,619 training sets and 2,007 validation sets, with a training-to-validation set ratio of around 7:1, or one-fifth of the overall dataset. The relevant code and dataset are publicly available at https://github.com/ZhaoHe1023/Improved-YOLOv4.

## 4. Designs for experiment

### 4.1. Experimental settings

All experiments in this thesis were performed on a computer equipped with a 5-core Intel(R) Xeon(R) Silver 4,210 CPU running at 2.20 GHz and an RTX 3,090 graphics card. Each algorithm was executed in the experimental environment using PyTorch 1.8.1, Python 3.8, and Cuda 11.1. In the training of the proposed model on the BDD-IW dataset, the input picture size is 512 × 512, the batch size is 16, the learning rate is 0.01, and the IoU detection threshold is 0.5. One hundred training rounds have been completed. To allow the final convergence of the detector on real-world data and without the error introduced by Mosaic, the model is supplemented with Mosaic and Mixup data for the first 85 training rounds and then switched off for the remaining 15.

### 4.2. Evaluation indicators

To objectively and reliably evaluate the detection performance of the proposed strategy, the experiments in this work employ the most frequent metric for target identification in studies: mean Average Precision (mAP). In this subsection, the mAP is explained.

Comparing the intersection and concurrence ratio (IoU) of ground truth and prediction with the threshold value allows the classification of the prediction results into four groups. False Positive (FP) is the positive sample of inaccurate prediction; True Negative (TN) is the negative sample of correct prediction, and False Negative (FN) is the negative sample of incorrect prediction. Table 1 depicts the confusion matrix.

**Table 1:** The confusion matrix.

	**N (Negative)**	**P (Positive)**
F (False)	FN	FP
T (True)	TN	TP

The number of FN, FP, TN, and TP can be empirically determined in the prediction process. Then, Equations (7), (8) yield Precision and Recall, respectively. Precision is the ratio of true cases (TP) to all positive cases (TP and FP) based on the model's evaluation. The recall is the proportion of correctly identified positive cases (TP) relative to all positive cases in the dataset (TP+FN).


(7) $$\begin{array}{l} Precision=\frac{TP}{TP+FP} \end{array}$$

(8) $$\begin{array}{l} Recall=\frac{TP}{TP+FN} \end{array}$$

It is not rigorous to evaluate the model's performance by Precision and Recall alone. In some extreme cases, the results of the two metrics on the model may be contradictory. Therefore, it was further analyzed by AP (Average Precision) and mAP (mean Average Precision). AP is the area enclosed by the P-R curve consisting of Precision and Recall. AP denotes a class's accuracy, mAP denotes all classes' average accuracy, and the mathematical formula precision is shown in Equations (9), (10).


(9) $$\begin{array}{l} AP=\int P\left(R\right)dR \end{array}$$

(10) $$\begin{array}{l} mAP=\frac{1}{C}\overset{C}{\underset{j}{\sum }}AP_{j} \end{array}$$

It is worth noting that mAP0.5 denotes the mAP at an IoU threshold of 0.5. mAP0.5:0.95 denotes the average mAP at different IoU thresholds (from 0.5 to 0.95, in steps of 0.05).

### 4.3. Quantitative evaluation

This subsection validates the target detection performance of the proposed method by the above-mentioned evaluation metrics such as AP50, mAP50, and FPS.

Table 2 shows the roadmap based on YOLOv4 improvements to verify the gain effect of the introduced improvement modules on the original method. Observing the data in Table 2, the improved module presented in this paper can make the algorithm perform better detection performance.

**Table 2:** Roadmap of the proposed method.

**Methods**	**mAP50 (%)**
YOLOv4 baseline	54.5%
+ Improved FPN	55.55% (+1.05%)
+ Anchor-free (SimOTA)	59.35% (+3.8%)
+ Decoupled head	59.75% (+0.4%)
+ Strong data augmentation	60.35% (+0.6%)
Proposed method	60.35%

**Figure 9:**
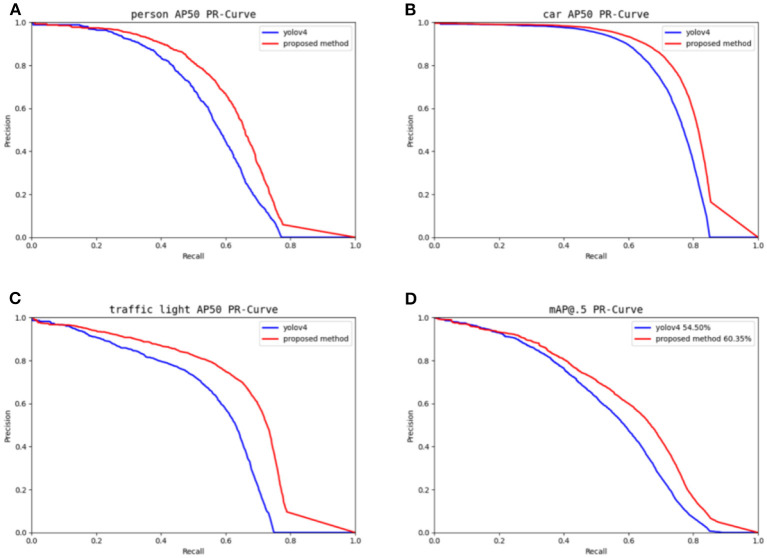
Main categories and overall P-R curves.

Figure 9 illustrates how the P-R curves are shown against YOLOv4, given that the BDD-IW data set contains three everyday objects: person, car, and traffic light. The area of the P-R curve is the AP value of the class and is a crucial parameter for assessing the output of the target detection algorithm. Figure 7 demonstrates that the P-R curves of the method described in this paper are centered on YOLOv4, demonstrating the success of the suggested work.

To objectively validate the target identification performance of the proposed method in this research, the suggested method is compared to some advanced methods on the BDD-IW dataset under identical settings. Among these techniques are Faster R-CNN (Girshick, 2015), SSD (Liu et al., 2016), YOLOv3 (Redmon and Farhadi, 2018), YOLOv4 (Bochkovskiy et al., 2020), YOLOv5 (Ultralytics), YOLOv6 (Li et al., 2022), YOLOX (Ge et al., 2021b), RT-YOLOv4 (Wang R. et al., 2021), TPH-YOLOv5 (Zhu et al., 2021), and PPYOLOE (Xu et al., 2022). The BDD-IW dataset comparison findings are displayed in Table 3.

**Table 3:** Comparison results of each method on the BDD-IW dataset.

**Methods**	**Person (%)**	**Car (%)**	**Bus (%)**	**Truck (%)**	**Bike (%)**	**Traffic light (%)**	**Traffic sign (%)**	**mAP50 (%)**	**FPS**	**Input size (pixels)**
Faster R-CNN	-	-	-	-	-	-	-	55.6	-	1,333 × 800
SSD	-	-	-	-	-	-	-	34.7	27.64	512 × 512
YOLOv3	47.8	69.0	44.1	51.0	26.0	46.9	48.9	47.7	43.57	512 × 512
YOLOv4	55.2	73.4	49.4	56.4	**34.0**	55.4	57.8	54.5	64.9	512 × 512
YOLOv5	54.5	73.2	52.3	57.2	32.7	55.0	56.1	54.4	72.21	512 × 512
YOLOv6	-	-	-	-	-	-	-	49.5	56.37	512 × 512
YOLOX	58.5	76.7	54.5	59.5	31.6	62.0	60.4	57.6	52.60	512 × 512
RT-YOLOv4	55.6	73.5	51.5	58.0	34.4	56.3	57.9	55.3	31.50	512 × 512
TPH-YOLOv5	51.0	71.8	45.4	52.9	26.1	55.2	57.1	51.4	35.10	512 × 512
PPYOLOE	52.9	72.7	46.4	53.5	28.8	56.5	58.9	52.7	47.12	512 × 512
Proposed work	**62.0**	78.8	**57.6**	**60.5**	33.6	**64.9**	**64.9**	**60.3**	69.40	512 × 512

FPS, frames per second.

The bold values indicate the best value in the same group comparison.

The following conclusions can be drawn from Table 3's data: First, on the BDD-IW dataset, YOLOv4 outperforms the one-stage models YOLOv3 and YOLOv5 in terms of detection accuracy, particularly for the most common person, automobile, and traffic light. YOLOv4 features an improved balance between detection speed and detection precision. Consequently, this comparison confirms the previous assertion that YOLOv4 is an excellent base method. Second, the proposed strategy greatly enhances each category's detection accuracy. The detection precision of the suggested method for easily concealed things, such as bicycles, is just 0.4 percent lower than that of YOLOv4 and ranks second. The proposed method topped all other categories of detecting techniques. Finally, the proposed technique has an mAP value of 60.3%, which places it top among all methods evaluated. The proposed approach yields an mAP of 2.7% more than YOLOX and 5.8% greater than the traditional YOLOv4. In addition, the detection efficiency of each detector processing the BDD-IW dataset can be seen in Table 3, and the proposed method achieves a detection speed of 69.4 FPS, which is better than the other methods except YOLOv5. Of course, the recognized advantage of the YOLOv5 algorithm is the higher detection speed caused by the lightweight network structure (Ultralytics, 2020), and the detection precision of the proposed method still has an advantage. The real-time detection speed required for autonomous driving is related to the detection image size, and according to previous studies (Hu et al., 2018; Zhao et al., 2019; Wang R. et al., 2021), when the input image size is 512 × 512 pixels or higher, the detection speed of the detector must reach more than 30 FPS in order to achieve higher real-time detection results.

In summary, the proposed method has improved the detection speed and precision compared to the original method.

### 4.4. Qualitative evaluation

**Figure 10:**
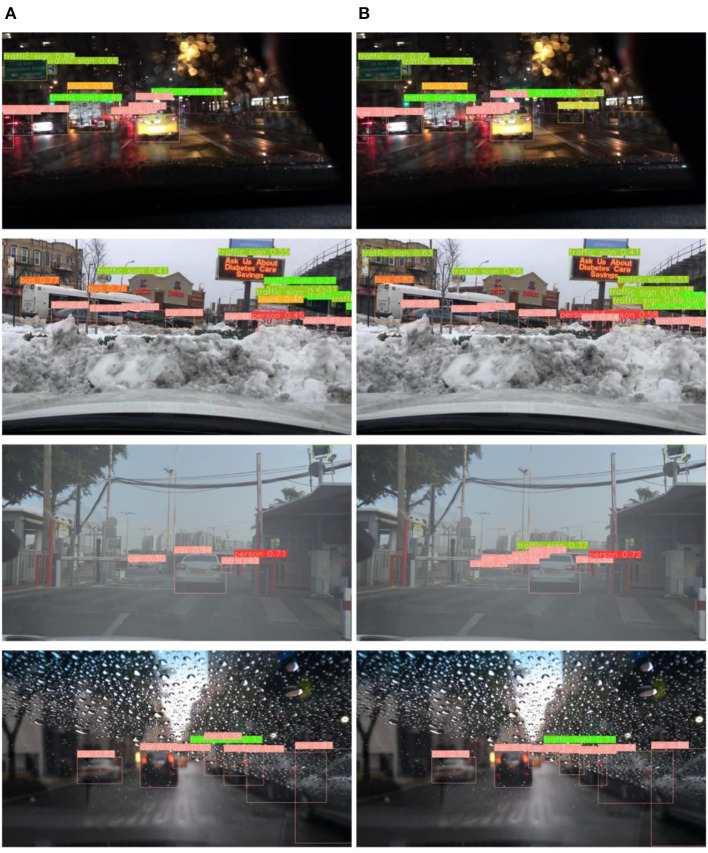
**(A)** YOLOv4 inference results. **(B)** Proposed work inference results.

On the BDD-IW dataset, Figure 10 compares the detection results of YOLOv4 with the proposed algorithm, where (a) is the detection effect of the YOLOv4 algorithm and (b) is the detection effect of the proposed method. In the detection effect graph, a comparison between the YOLOv4 method and the method proposed in this paper reveals that the YOLOv4 method loses vehicle feature information after the multi-layer convolution operation, resulting in the leakage of small-scale vehicles and obscured vehicles during the detection process. As shown in the first row, the proposed method detects the small-scale targets of bike and traffic sign on the right side of the image in the dim environment compared to YOLOv4. And the detection performance of the proposed method is obviously better in snow, fog, rain and other adverse weather conditions for obscured or blurred vision targets. Also, each comparison image demonstrates that the proposed method identifies more accurately.

In conclusion, the algorithm proposed in this paper greatly enhanced precision compared to the original YOLOv4 target identification algorithm and other advanced models of the target detection algorithm. It is more suited for detecting small and obscured targets in inclement weather conditions, enhancing driving stability and effectiveness.

## 5. Conclusion and future work

For autonomous driving and road safety, achieving more precise vehicle target detection in inclement weather situations is of tremendous scientific importance. This research provides an enhanced method based on YOLOv4 to boost the performance of target detection under inclement weather conditions to improve the precision and speed of target detection. By comparing the BDD-IW inclement weather image dataset, experimental comparisons are conducted. The prediction precision of this paper's proposed method is greater than that of some well-known target detection systems. Moreover, compared to the conventional YOLOv4 method, the mAP of the method suggested in this study is enhanced by 5.8%. The proposed method performs well in target detection, especially for vehicle target detection in inclement weather conditions. As can be seen from the quantitative and qualitative analysis of detection precision and detection speed, the method effectively avoids undetectable or erroneous detection of vehicles or road objects in bad weather conditions during smart driving. In addition, the proposed method has achieved the requirement of real-time target detection. The higher detection speed helps the intelligent driving system to make the next decision faster and improves the safety of unmanned driving.

However, there are still some problems that need to be further explored. Although the speed of detection has been increased compared to the original method, there is still potential for improvement. The precision of target occlusion needs to be improved. The target cannot be detected correctly with high probability in the occlusion situation, so how to further improve the recognition rate of the vehicle is a worthy research direction in the later stage. Regarding the practical application of target detection, the algorithm researched in the laboratory is tested using GPU training and inference, while there is no GPU on the real self-driving detection platform, so the portability of the model needs further study.

## Data availability statement

The datasets presented in this study can be found in online repositories. The names of the repository/repositories and accession number(s) can be found in the article/supplementary material.

## Author contributions

RW: conceptualization. HZ: methodology, software, and validation. YD: formal analysis. GL: data curation. ZX: writing—review and editing. YZ and HL: supervision. All authors contributed to the article and approved the submitted version.
